# Spontaneously-forming spheroids as an *in vitro* cancer cell model for anticancer drug screening

**DOI:** 10.18632/oncotarget.4013

**Published:** 2015-06-18

**Authors:** Maria A. Theodoraki, Celso O. Rezende, Oraphin Chantarasriwong, Adriana D. Corben, Emmanuel A. Theodorakis, Mary L. Alpaugh

**Affiliations:** ^1^ Department of Biology, Arcadia University, Philadelphia, PA, USA; ^2^ Department of Chemistry and Biochemistry, University of California - San Diego, La Jolla, CA, USA; ^3^ Department of Chemistry, Faculty of Science, King Mongkut's University of Technology Thonburi, Bangkok, Thailand; ^4^ Department of Pathology, Memorial Sloan Kettering Cancer Center, New York, NY, USA; ^5^ Department of Surgery, Memorial Sloan Kettering Cancer Center, New York, NY, USA

**Keywords:** drug screening, lymphovascular embolus (LVE), natural products, Garcinia xanthone motif (CGX), breast cancer

## Abstract

The limited translational value in clinic of analyses performed on 2-D cell cultures has prompted a shift toward the generation of 3-dimensional (3-D) multicellular systems. Here we present a spontaneously-forming *in vitro* cancer spheroid model, referred to as spheroids*^MARY-X^*, that precisely reflects the pathophysiological features commonly found in tumor tissues and the lymphovascular embolus. In addition, we have developed a rapid, inexpensive means to evaluate response following drug treatment where spheroid dissolution indices from brightfield image analyses are used to construct dose-response curves resulting in relevant IC_50_ values. Using the spheroids*^MARY-X^* model, we demonstrate the unique ability of a new class of molecules, containing the caged *Garcinia* xanthone (CGX) motif, to induce spheroidal dissolution and apoptosis at IC_50_ values of 0.42 +/−0.02 μM for gambogic acid and 0.66 +/−0.02 μM for MAD28. On the other hand, treatment of spheroids*^MARY-X^* with various currently approved chemotherapeutics of solid and blood-borne cancer types failed to induce any response as indicated by high dissolution indices and subsequent poor IC_50_ values, such as 7.8 +/−3.1 μM for paclitaxel. Our studies highlight the significance of the spheroids*^MARY-X^* model in drug screening and underscore the potential of the CGX motif as a promising anticancer pharmacophore.

## INTRODUCTION

The majority of studies on cellular responses to extracellular stimuli have relied on the use of established, immortalized cells grown as 2-dimensional (2-D) monolayers. Albeit tremendously useful for cell biology studies, these 2-D cultures fall short in recapitulating the complex native environment of living tissue and thus have limited value in clinic. For instance, neighboring cells as well as the extracellular matrix (ECM) are known to induce epigenetic factors that regulate key events of the cell cycle, such as proliferation, migration and cell death [1, 2]. Most significantly, 2-D monolayer cultures fail to accurately represent the complex heterogeneous cell-cell interactions as well as the effects of the microenvironment (e.g. hypoxia) typically found in multi-cellular tissue that can modulate gene expression and alter the molecular signaling profile of specific populations of cells [3, 4]. These observations have led to the pursuit of three-dimensional (3-D) cell cultures that have the potential to improve the physiological relevance of cell-based studies and increase the successful translation of cell-based drug screening in the discovery of new therapeutics [5–8].

Multi-cellular 3-D systems represent a more relevant model of living tissue and can also provide valuable insights into processes that govern cancer progression, metastasis and drug resistance [6, 9]. At present, methods that establish 3-D cell models include but are not limited to hanging drop, liquid overlays and microfabrication (e.g. cellular gel encapsulation) [5, 10–14]. In general, these methods generate artificial environments that drive cells that under normal conditions would grow as a traditional 2-D culture, to form 3-D spheroid-like structures. The goal is to create multi-cellular 3-D models that acquire a more tissue-like phenotype [6]. Although these methods provide a marked improvement in comparison to 2-D cultures, several factors, such as culture longevity that may fail to attain tissue-like phenotype, reproducibility of culture conditions and spheroid size, limit their translational utility as clinically relevant models. Additionally, these methods often generate a limited number of spheroids (e.g. the hanging drop method generates 1 spheroid/well [14]) at a substantial cost, making these systems impractical for high-throughput drug screening and development.

Development of *in vitro* models that more closely resemble living tissue is extremely important in order to decipher cell-cell interactions and signaling especially within the complex tumor tissue environment. Additionally, metastatic disease is the single most crucial reason for morbidity due to cancer and is typically defined by the presence of lymphovascular emboli (LVE), i.e. clumps of cancer cells found within the lymphatics and/or blood vessels [15]. Cancer cells that constitute either the LVE or the tumor are governed by external pressures that vary depending on cell location and microenvironment. Such heterogeneous masses have decreased sensitivity to chemotherapeutics and, in fact, LVE are viewed as reliable markers for recurrent breast cancer that is resistant to radio- and chemotherapy [16, 17]. 3-D culture models best recapitulate the biological and biochemical heterogeneity of the *in vivo* embolus/intratumoral cellular mass where, due to external constraints, oxygen, pH and nutritional gradients develop that can significantly impact response to therapeutic agents [18–20]. Therefore, employing 3-D models in drug development is imperative for successful translation of anticancer therapeutics to the clinic.

Here we report on a spontaneously-forming spheroid model, referred to as spheroids*^MARY-X^*, that has distinct advantages over induced 3-D models. Namely, this model has an innate ability to form compact, tight spheroids, where the compaction conveyed by molecular determinants has been proven to contribute to metastatic progression and efficiency [21, 22]. These spheroids mimic both the *in vivo* metastasis (i.e. LVE) [23, 24] and the intratumoral biological/biochemical complexities. In addition, the spheroids*^MARY-X^* are not limited in yield and, in conjunction with multi-well plate analyses, could provide a high-throughput (HTP) platform with predictive value for anticancer drug screening and development [25]. We validated the use of spheroids*^MARY-X^* as a new platform for the screening of various small molecule therapeutics. Significantly, we demonstrate for the first time that synthetic compounds derived from the caged *Garcinia* xanthones (CGX) family of natural products [26] display potent cytotoxic effects while Federal Drug Administration (FDA)-approved therapies for both solid and blood-borne tumor types fail to elicit a response.

## RESULTS

### Spontaneous formation, morphology and size selection of spheroids*^MARY-X^*

**Figure 1:**
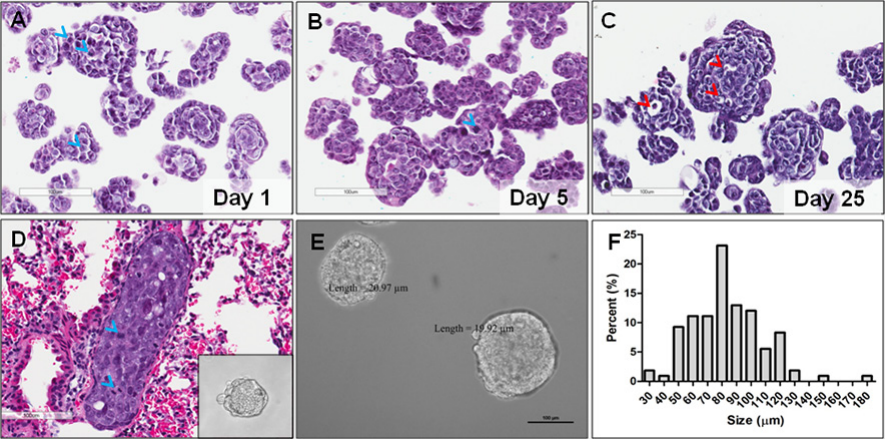
Spheroids*^MARY-X^* of primary tumor explant and lung metastasis **(A)** Spheroids are composed of individual cells with large, fit nuclei as evidenced by mitotic events (blue arrow heads); **(B, C)** Spheroids remain viable in tissue culture up to and beyond 25 days with few visible apoptotic events (red arrow heads); **(D)** Metastatic lung emboli are composed of individual cells with large, fit (i.e. mitotic, blue arrow head) nuclei; **(D)** insert and **(E)** *In vitro*, compaction of the individual cells results in spheroids with well-circumscribed edges of both the tumor and lung, respectively (**A–E**, bar 100 μm); **(F)** Spheroid preparations used in drug screens range in size from slightly ≤ 40 μm to slightly ≥ 100 μm.

MARY-X, *in vitro*, is a primary cellular derivative from tumor explants. These tumor cells spontaneously form tight, compact aggregates of cells termed spheroids^*MARY-X*^ (Figure 1A–1C, 1D insert and 1E). Comparable to human inflammatory breast cancer (IBC) emboli, a persistent over-expression of an intact E-cadherin/α, β-catenin axis mediates the compaction of both *in vitro* and *in vivo* spheroids*^MARY-X^* and tumor emboli, respectively. This persistent over-expression is maintained throughout metastatic progression allowing for spheroids*^MARY-X^* derivation from both the primary tumor and lung metastasis (Figure 1D insert and 1E). For practical purposes (i.e. spheroid quantity), spheroid derivation for drug screening was carried out on spheroids*^MARY-X^* obtained from the primary tumor. The spheroids*^MARY-X^* range in size from as small as 20 μm to as large as 600 μm in diameter. For this study, spheroids*^MARY-X^* ranging in size from ~40 μm to ~100 μm were partitioned as previously reported [27] and used for all drug screening (Figure 1F and Supplementary Figure S1, Supplementary Data). Drug screens were typically performed within 5 days of the spheroids*^MARY-X^* preparation. However, these spheroids*^MARY-X^* remain viable in culture as evidenced by fit nuclei displaying mitotic activity in spheroids on day 1 as well as in day 5 and 25 (Figure 1A, and 1C) with few apoptotic events seen only in larger spheroids*^MARY-X^* at day 25 (Figure 1C).

### Pathophysiological gradient of spheroids*^MARY-X^*

**Figure 2:**
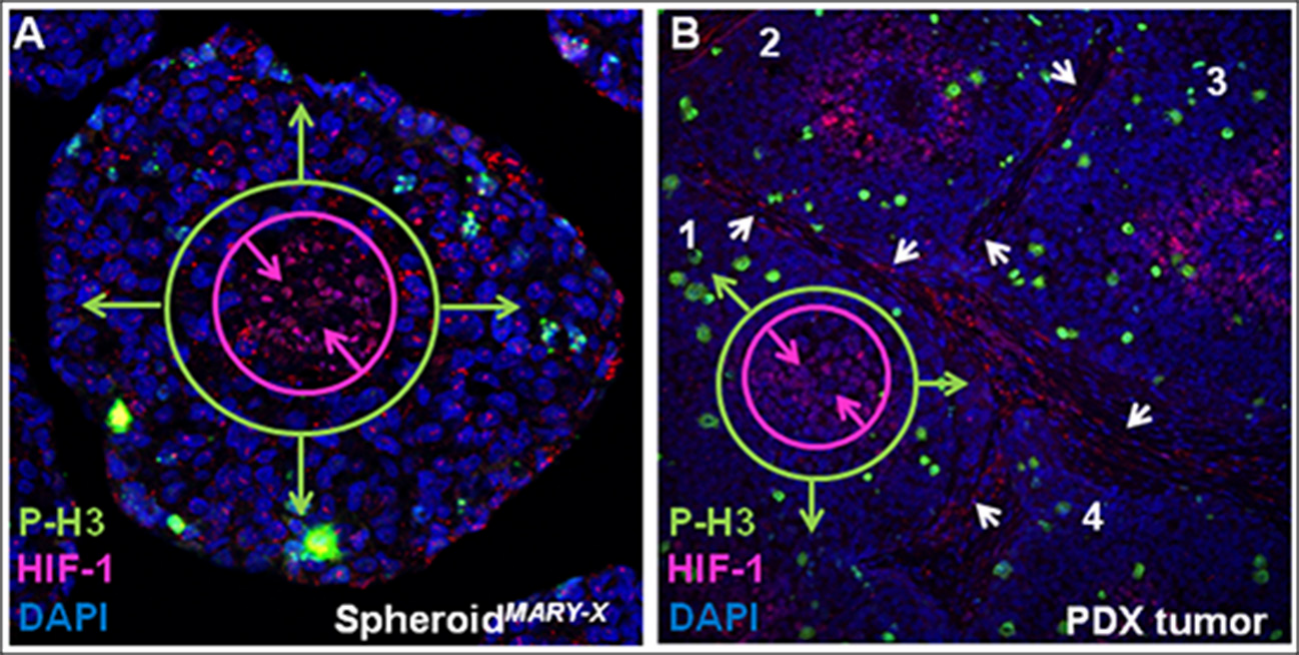
Pathophysiological gradient of spheroids*^MARY-X^* **(A)** *In vitro*, spheroid cellular mass show differential areas of hypoxia and proliferation; **(B)** The IBC PDX tumor (tumor emboli 1–4) show distinct areas of high proliferation and hypoxia within each tumor embolus. (**A & B 63X** magnification).

The *in vitro* spheroids*^MARY-X^* (Figure 2A) are under physiological constraints, such as diffusion, similar to the *in vivo* solid tumor and/or lymphovasular embolus (Figure 2B). A region located towards the periphery of the spheroid stains positive with phospho-histone 3 (P-H3), a mitotic marker identifying a proliferative cell subpopulation. Cells that are centrally-located stain positive for the hypoxia-inducing factor-1 (HIF- 1) with a negative P-H3 status (Figure 2A) and are, therefore, in a quiescent cellular state. Therapeutics (e.g. chemo/irradiation) that rely on a high cellular proliferation status would be ineffectual on those cells found in the hypoxic region of the cellular mass. Significantly, the spheroids*^MARY-X^* recapitulate the biological/biochemical complexity found *in vivo* (Figure 2B). In this 2 cm in diameter IBC PDX tumor, all four emboli (Figure 2B; stromal boundary demarcated with white arrows; 1–4) display a peripheral P-H3 positive cell population with a more centrally-located hypoxic region void of P-H3 positive cells (Figure 2B; emboli 1 annotated only). These data are consistent with immunohistochemical analyses of spheroids*^MARY-X^* with the proliferative marker, Ki67 and hypoxic marker CAIX, (Supplementary Figure S2, Supplementary Data), where highly proliferative cells are found exclusively on the outer perimeter of spheroids*^MARY-X^*. The spheroids*^MARY-X^* contain pathophysiological gradients consistent with the native *in vivo* environments of the solid tumor and lymphovascular embolus and therefore provide a very relevant model in the screening of drugs in development.

### Federal drug administration (FDA)-approved anticancer therapeutic drug screen

**Figure 3:**
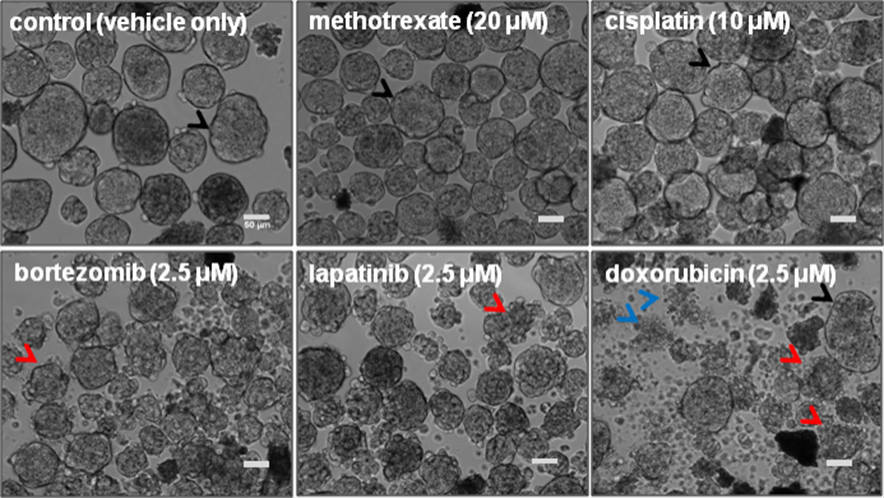
FDA-approved anticancer drugs screened using spheroids*^MARY-X^* Methotrexate and Cisplatin show no response retaining well-circumscribed spheroid edges (black arrows) identical to the control. Low to moderate with slightly distorted spheroid edges (red arrows) response is seen in bortezomib and lapatinib, whereas doxorubicin displays a mixed response where distorted (red arrows) and well-circumscribed spheroids (black arrows) coexist as well as a single cell population (blue arrows) a sign of complete response. (bar 50 μm)

To determine how standard of care drugs would perform in a spheroids*^MARY-X^* screen, several FDA-approved drugs known to be effective in solid tumor types as well as blood-borne malignancies, were evaluated. Spheroids were added to a multi-well plate (~30–50 spheroids/well) and then treated with vehicle only (DMSO) and increasing therapeutically-relevant drug doses as follows: bortezomib, lapatinib and doxorubicin (0–2.5 μM) [28–32], cisplatin (0–10 μM) [32] and methotrexate (0–20 μM) [33]. Therapeutically-relevant is defined as a drug dose that is either recorded in literature [32, 34–37] or previous experiments as being the effective *in vitro* dose as determined in 2D cancer models. Following a 24-hr treatment period each well underwent analysis to assess dissolution of the spheroids*^MARY-X^* (Figure 3). The induction of apoptosis correlates with the loss of well-circumscribed edges of the usually tight, compact spheroids*^MARY-X^* i.e. dissolution is consistent with cell death of the spheroid/embolus [27]. Both methotrexate and cisplatin showed no drug response as the spheroids*^MARY-X^* maintained well-circumscribed edges comparable to the control (Figure 3, upper panels, black arrows) indicative of spheroid viability. Both bortezomib and lapatinib showed a slight response where spheroid edges become slightly distorted (Figure 3, lower left and middle panels, red arrows). This is in contrast to doxorubicin which showed a mixed response in the treatment of spheroids*^MARY-X^*, where spheroids with well-circumscribed edges (Figure 3, lower right panel, black arrows) coexist with spheroids with significantly distorted edges (Figure 3, lower right panel, red arrows) as well as single cell populations (Figure 3, lower right panel, blue arrows), indicative of complete response. To determine whether the limited response was due to either doxorubicin only targeting proliferative cells found predominantly on the outer periphery of the spheroids*^MARY-X^* or whether drug penetration contributed to the outcome, the spheroids were treated with the highest dose at 2.5 μM for 3 hrs and 24 hrs. Doxorubicin has an intrinsic fluorescence [38], and therefore, cell-cell distribution and drug penetration within the spheroid was determined using confocal microscopy with z-stack analysis. Orthogonal image slices minus volume of both the 3 hr (Supplementary Figure S3, Supplementary Data) and 24 hr (data not shown) treatment periods displayed drug penetration throughout the entire spheroid. Data indicates that there is no limitation to drug penetration since doxorubicin is found throughout the spheroids*^MARY-X^* as early as 3 hrs and remained present up to the 24 hr treatment period. Overall, only one of the five FDA-approved drugs, doxorubicin (Table S1, Supplementary Data) showed potential efficacy in treatment of a relevant model of breast cancer and metastatic disease. Additionally, tests with PU-H71, a potent Hsp90 inhibitor that is presently in clinical development showed no response when used in the spheroids*^MARY-X^* model (Supplementary Figure S4, Supplementary Data), even though in preclinical analysis this drug was found to initiate complete response in several triple-negative breast cancer 2-D culture models.

### Drug screen using *in vitro* spheroids*^MARY-X^*

**Figure 4:**
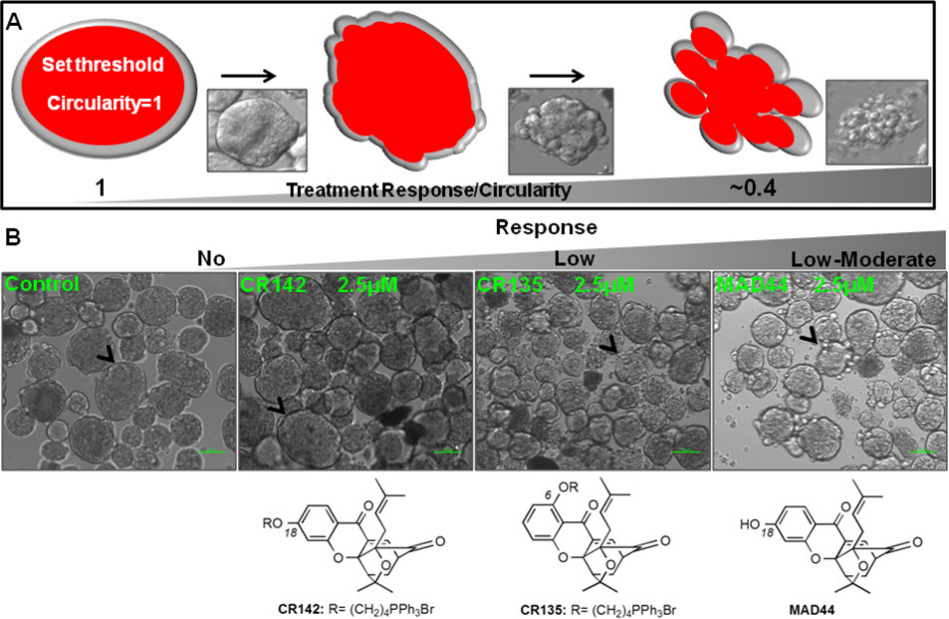
Drug screening using spontaneously-forming spheroids*^MARY-X^* **(A)** Images from a brightfield microscope (panel insets) are analyzed on ImageJ using an image analysis program where ‘1’ signifies a perfect circle (i.e. intact spheroid) and numbers approaching ‘0’ signify loss of circularity (i.e. dissolute spheroid). Note that ‘0’ signifies a straight line and can never be achieved in image analysis of spheroids given the innate circularity of individual cells. **(B)** Deviations from a well-circumscribed spheroid edge (Control, black arrow heads) are ranked from no (‘no’ response; CR142) as well low (‘low’ response; CR135) to moderate (‘low-moderate’; MAD44) response where the formerly well-circumscribed spheroid edge is slightly distorted. (bar 100 μm)

**Figure 5:**
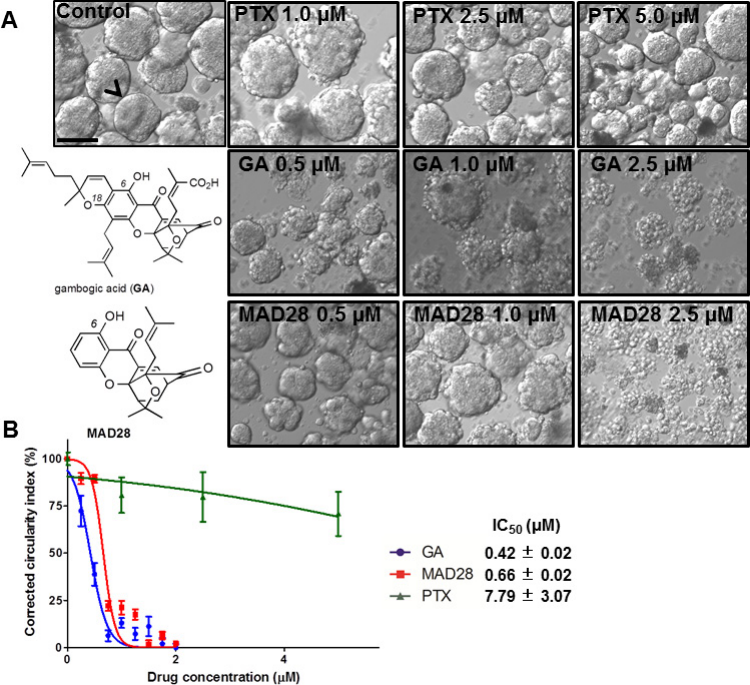
Dose response curve rendering IC_50_ values of drugs using spontaneously-forming spheroids*^MARY-X^* drug screen **(A)** Gambogic acid (GA) and MAD28 display ‘complete’ response as there is total dissolution of the formerly intact spheroid with well-circumscribed edges to the single cell state **(B)** Dissolution indices are determined through image analysis and used to construct a dose-response curve and subsequent IC_50_ of drugs that displayed response as compared to the standard of care of metastatic breast cancer (i.e. Paclitaxel). (bar 100 μm)

The spontaneously-forming spheroids*^MARY-X^* provide an efficient high-throughput platform to screen for efficacy of drugs in development. For this initial investigation focused predominantly on cytotoxicity, spheroids*^MARY-X^* were seeded either sparsely (~30–50/well) for image analysis or more densely (~100/well) in a replicate plate for further analysis of induction of apoptosis. As the standard treatment of metastatic breast disease and several solid tumor cancer types, paclitaxel at 1.0, 2.5 and 5.0 μM concentration was included as a control in all drug screens. Synthetic analogues of gambogic acid (GA), CR142, CR135, MAD44 and MAD28, as well as GA were used in this drug screen (Figure 4B and Figure 5A). Each well was treated with vehicle only (DMSO) and increasing doses of compound at 0.5, 1.0 and 2.5 μM concentrations, based on previously reported drug doses of similar structure [39] (Figure 4B and Figure 5A). Only those compounds showing promising response as indicated by a dose-dependent response underwent further image analysis to determine response (i.e. IC_50_).

**Scheme 1:**
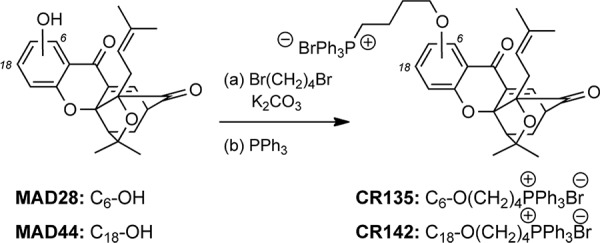
Reagents and conditions for the synthesis of CR135 and CR142 **(a)** 1, 4-dibromobutane (5 equiv.), K_2_CO_3_ (2 quiv.), DMF, 25–80°C, 8–16 h; **(b)** PPh_3_ (5 equiv.), CH_3_CN, 150°C, microwave irradiation, 2 h; 58% for CR135, 83% for CR142.

Following a 24-hr treatment period each well underwent image analysis to assess dissolution of the spheroids*^MARY-X^*. A well-circumscribed spheroid edge or periphery as seen in the control spheroids*^MARY-X^* was indicative of no drug response, whereas sensitivity is measured by a deviation from a well-circumscribed edge (Figure 4A). The response spectrum of analogs tested ranged from ‘no’ to ‘low - moderate’ (Figure 4B) with the exception of MAD28 which, comparable to GA, exhibited a ‘complete’ response (i.e. total spheroid dissolution) (Figure 5A). This response spectrum can provide sufficient qualitative information regarding a small molecule structure activity profile. Namely, it is worth noting that GA and MAD28, two CGX analogs containing a C_6_-phenol group induce a ‘complete’ response. Under identical conditions, MAD44, a structural isomer of MAD28 containing a phenol group at C_18_ induces a ‘low-moderate’ response. Moreover, functionalization of both MAD28 and MAD44 at the pendant phenol group with a triphenyl phosphonium side chain attenuates the compound activity (Scheme 1). Taken together, the results attest to the biological significance of the β-hydroxyketone functionality of MAD28 and GA. Interestingly, paclitaxel, a drug used in clinic for treatment of metastatic breast cancer, was virtually ineffective (Figure 5A). To get a more accurate assessment of GA and MAD28 drug response an additional screen with numerous drug concentrations at 0.25 μM increments (0.25–2.0 μM) was performed (Supplementary Figure S5, Supplementary Data). Comparable to the first screen, GA began to display response at a lower drug concentration, 0.50 μM, in comparison to MAD28 which began to display response at 0.75 μM (Supplementary Figure S5, red arrows, Supplementary Data). Both GA and MAD28 showed complete response at 1.75 μM (Supplementary Figure S5, Supplementary Data). Because the induction of apoptosis correlates with the loss of the well-circumscribed edges of the usually tight, compact spheroids*^MARY-X^* (i.e. dissolution is consistent with cell death of the spheroid/embolus), the IC_50_ is determined by applying an image analysis program that measures percent dissolution indices (i.e. circularity of well circumscribed edges of intact spheroid vs. dissolute single cells as shown in Figure 4A) and using these data to prepare a dose-response curve. Using this method, the calculated IC_50_ value for paclitaxel was found to be 7.79 +/−3.07 μM (Figure 5B). On the other hand, the IC_50_ values of GA and MAD28 were found to be 0.42 +/−0.02 μM and 0.66 +/−0.02 μM, respectively (Figure 5B). Thus, the dissolution indices method allows for the acquisition of quantitative information on the compound activity, further streamlining the use of the spheroids*^MARY-X^* assay as a drug screening platform.

### Dissolution indices vs. standard viability assay

**Figure 6:**
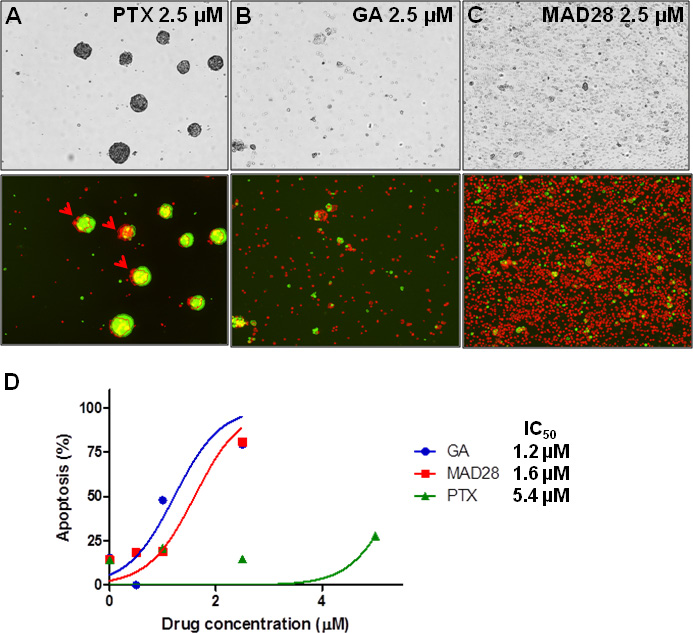
Dual fluorescence viability assay of treated spheroids*^MARY-X^* **(A)** Paclitaxel is ineffective at producing a ‘complete’ response by inducing apoptosis of the spheroid cells predominantly located on the outer periphery (red arrow head). **(B)** Gambogic acid (GA) and **(C)** MAD28 display ‘complete’ response as there is total dissolution of the formerly compact spheroid with well-circumscribed edges to the single cell state. **(D)** Dose-response curve results in comparable pattern of response (i.e. IC_50_ of drugs).

In an effort to validate response data acquired by image analysis (i.e. dissolution indices) an alternate method, traditionally used to determine and quantify cell viability, was performed on a replicate drug screen. This drug screen reproduced drug dose parameters of paclitaxel (PTX), gambogic acid (GA) and MAD28. Viability was measured by dual fluorescence analysis of propidium iodide (PI) and acridine orange (AO). Following the 24-hr treatment period, spheroids were analyzed using the Cellometer K2 in the dual-fluorescence viability assay. The nuclear stains AO and PI were added to the control (vehicle only) and treated spheroids*^MARY-X^*, where AO stains both live and dead (green) cells and PI stains only dead (orange) cells with compromised membranes. Fluorescent images presented here are one of eight random fields captured for the viability analyses. Comparable to the previous results, PTX showed little if any activity predominately on the outer periphery of the spheroid (Figure 6A red arrows), whereas both GA and MAD28 displayed complete response as seen by the total dissolution of the formerly compact spheroids*^MARY-X^* to a single cell state where the majority of cells are non-viable (dead) (Figure 6B and 6C). The IC_50_ of each drug as calculated from the dose-response curve was 1.2, 1.6 and 5.4 μM for GA, MAD28 and PTX respectively (Figure 6D), showing a pattern of response comparable to those obtained from the dissolution indices.

## DISCUSSION

It is now evident that traditional 2-dimensional (2-D) monolayer cell cultures are incapable of recapitulating the heterogeneous and dynamic parameters of the multicellular tumor tissue and lympovascular embolus, a marker of cancer aggressiveness, recurrence, metastatic progression and most significantly, therapeutic failure. The limitations of traditional 2-D monolayer cell cultures to predict *in vivo* response are particularly important in the drug-screening process and may account for the high failure rate and rising costs of drug discovery [40, 41]. On the other hand, the use of animal models in drug development are time-consuming, expensive and often do not accurately reflect human cancer biology [42, 43]. The gap between 2-D cultures and animal models could be bridged by the development of multidimensional screening methods that, in combination with advances in digital imaging, promise to exponentially increase our understanding on cellular behavior and revolutionize traditional drug discovery. Yet, at present most 3-D multicellular spheroids are typically developed from formerly traditional 2-D monolayer cell culture, providing only pseudo-representations of multicellular tumor tissue. This is in stark contrast to the spheroids*^MARY-X^* model, where innate molecular determinants are directly responsible for the spontaneously-forming tight, compact spheroids. The spontaneous formation results in high spheroid yield at a relatively low cost, a distinct advantage over existing 3-D spheroid-generating methods.

The microenvironment dictates pathophysiological parameters of both the tumor tissue and lymphovascular embolus. The spheroid morphology recapitulates these parameters rendering a cellular mass whereby each cell is under unique selective pressure dependent on the location. Each cell or cell population undergoes molecular changes in response to unique environmental (i.e. nutritional and oxygen supply) and selective pressures resulting in pathophysiological gradients where zones of highly proliferative and quiescent/dormant cells (hypoxic region) are established [9]. The spheroids*^MARY-X^* contain a peripheral zone where highly proliferative cells reside as well as a population found within the spheroids*^MARY-X^* core of quiescent/dormant (i.e. non-proliferative) tumor cells as a result of hypoxic conditions. Although therapeutic resistance can be the result of several molecular factors, it is understood that dormant tumor cells are highly refractory to chemotherapeutics and can result in relapse and late-developing metastases [44]. To the contrary, 3D culture systems derived from traditional 2D cell lines fail to properly recapitulate this feature of *in vivo* tumor tissue (e.g. tumor cell dormancy), where 3–6 weeks of culture growth is needed for these multicellular tumor spheroids to reach 400–600 μm in diameter and even at this size, proliferative (Ki67 positive) cells either extend throughout the spheroid [45–48] or are sparsely located on the periphery and the spheroid itself, shows signs of central necrosis [49]. Developing improved drugs or drug delivery systems to circumvent tumor cell dormancy, as well as *in vitro* cell models with predictive value is critical in the success of treatment of metastatic disease and cancer in general. The spontaneously-forming *in vitro* spheroids*^MARY-X^* model has captured the biological/biochemical complexities of both the tumor tissue and lymphovascular embolus. The image analysis rendering dissolution indices used to generate dose response curves provide an initial, no cost, rapid and efficient means to screen multiple drug analogs or anticancer therapeutics. The model is also amenable to both colorimetric and fluorescent cell viability analyses highlighting the versatility of this high-throughput model system. These key features of the spontaneously-forming spheroids*^MARY-X^* provide a relevant model with rapid, efficient and clinically translatable data in drug design and development.

Screening of the library of FDA-approved drugs in the spheroids*^MARY-X^* highlights the possible predictive value of this model in contrast to traditional 2-dimensional (2-D) monolayer cultures, commonly used to determine drug potency prior to costly, laborious *in vivo* animal efficacy studies. These drugs were chosen because each target cancer-specific vulnerabilities such as DNA synthesis (cisplatin, doxorubicin and methotrexate [50–52]), epidermal growth factor receptor (EGFR) (lapatinib) [53] along with the nuclear factor (NF)-κB signaling pathway (bortezomib) known to be constitutively active in many cancer types [54]. Significantly, three of the five drugs used in this drug screen, namely methotrexate, doxorubicin and lapatinib, are specific for use in breast cancer [55]. Additionally, doxorubicin and lapatinib have been approved for treatment of metastatic or late stage breast cancer. At the high end of therapeutically relevant doses as reported in 2-D *in vitro* models [28–32], out of the 5 drugs tested, only bortezomib and lapatinib displayed a low response as indicated by a slightly distorted edge of the spheroids following treatment. On the other hand, treatment with doxorubicin elicited a mixed response where both intact as well as partially dissolute (i.e. single cell population) spheroids*^MARY-X^* coexisted. A possible interpretation of this mixed response, after eliminating drug penetration issues, is that doxorubicin is targeting proliferative cells that compose either smaller spheroids or the outer periphery of larger spheroids, thereby leaving behind tumor cells that are still in a predominantly dormant state. This is particularly alarming since after chemotherapy, the remaining dormant tumor cells often lead to resistance, relapse and metastatic disease. Overall, these FDA-approved drugs performed rather poorly in a model system that has captured the pathophysiological features of both the tumor tissue and the lymphovascular embolus.

As well as being a preclinical model of inflammatory breast cancer (IBC) [21], the most aggressive form of breast cancer [56–58], the spheroids*^MARY-X^* are also receptor status negative. Recently, a 2^nd^ generation Hsp90 inhibitor, PU-H71 was found to be efficacious in triple-negative 2D breast cancer models [59]. Therefore we decided that it was warranted to compare and evaluate a drug proven effective in comparable (i.e. triple negative) albeit non-IBC, 2D breast cancer models to our 3D model. This Hsp90 inhibitor, although found to be effective in triple negative 2D breast cancer models, failed to generate a response in the spheroids*^MARY-X^* 3D model.

We also used the spheroids*^MARY-X^* screening assay to evaluate compounds currently in preclinical development [39, 60]. Paclitaxel, a drug used in the treatment of breast cancer, was used as a control. Interestingly, this drug exhibited only a ‘no to low’ response at a 5.0 μM concentration. In contrast, gambogic acid treatment led to complete spheroid dissolution at a 1.75 μM concentration. MAD28, a synthetic analog of gambogic acid that contains its pharmacophoric motif (i.e. caged structure and the C8-β-hydroxyketone functionality), was found to have similar potency with complete spheroid dissolution at 1.75 μM. On the other hand, MAD44, a GA analog that contains the caged structure but lacks the β-hydroxyketone functionality induced moderate response at similar concentrations. We found that derivatizing the phenol group of these compounds with an alkylphosphonium side chain led to significant decrease of activity. Specifically, CR142, an alkylated derivative of MAD44, showed no response, while CR135, an alkylated derivative of MAD28, induced only moderate response. These data indicate that the spheroids*^MARY-X^* assay can be used to identify new anticancer leads as well as provide useful information on structure-function relationship studies.

Several criteria define a high-throughput screening platform, such as, (a) a multiwell format (96-well plate or greater), (b) assay volume (e.g. 96-well, 50–200 μl), (c) assay steps (10 steps including reagent additions, incubation time, plate transfer and reading), (d) assay time (between 5 min and 48 hrs), (e) reagent addition (4 maximum), and (f) detector (e.g. microplate imager) [61]. The spheroids*^MARY-X^* model as a drug screening platform meets and in many instances exceeds these criteria, namely the assay can be accommodated in a 96-well plate or greater, requires ≤ 5 assay steps and importantly, the drug screen can be performed in one multiwell plate without media replacement or exchange, and does not require additional molecular reagents to measure response. Most significantly, rapid measurement of response is essential in any high throughput drug screening model system [25, 61–63]. As previously determined the induction of apoptosis correlates with the loss of well-circumscribed edges of the usually tight, compact spheroids*^MARY-X^* where dissolution into a single cell population is consistent with cell death of the spheroid [27]. Dissolution indices are determined through simple brightfield image analysis of circularity (intact spheroid) vs. dissolute (single cell populations) data which were then used to plot dose response curves and subsequently calculate IC_50_ values. The IC_50_ values determined in this screen of library of drugs, was found to be 7.8 +/−3.1 μM for paclitaxel, 0.42 +/−0.02 μM for GA and 0.66 +/−0.02 μM for MAD28. These values were validated with the more commonly used dual fluorescence viability assay of acridine orange and propidium iodide. Comparable IC_50_ values were measured using the dual fluorescence assay. However, the dissolution indices approach, presented in this study, provides a more rapid and low tech means to measure response in the spheroids*^MARY-X^* model system.

In conclusion, we describe herein the use of spheroids*^MARY-X^* as a reliable platform for anticancer drug screening. Developed from an inflammatory breast cancer patient-derived xenograft, this model system has distinct advantages over other screening platforms since it creates 3-D cultures that (a) are spontaneously forming and thus do not require extraneous protocols to induce spheroidal morphology; (b) retain the parent tumor phenotype and accurately mimic both the *in vivo* metastasis (i.e. lymphovascular embolus) and the intratumoral biological complexities of the living tissue; (c) are high yielding at a low cost; and (d) amenable to high-throughput drug screening. Additionally, we have developed a rapid, quantitative means to measure drug response and calculate IC_50_ values using the dissolution indices. The obtained IC_50_ values correlate well to those derived from a dual fluorescence assay. Most significantly, using this screening platform we identified a new family of small molecules, based on the caged *Garcinia* xanthone (CGX) motif, which induces complete spheroid dissolution at submicromolar concentrations. Based on our results, the CGX motif represents a promising anticancer pharmacophore that can be used to treat both IBC, a cancer with no known targeted therapy, as well as all cancers that do not remain organ-confined (i.e. spheroids*^MARY-X^* mimic the lymphovascular embolus).

## MATERIALS AND METHODS

### MARY-X xenograft and *in vitro* spheroids

MARY-X is an inflammatory breast cancer (IBC) patient-derived xenograft (PDX) [21]. *In vivo*, the PDX model precisely captures the human IBC signature phenotype of extensive intravasation *in situ* of the lymphatic and blood vessels by tumor emboli. The IBC spheroids are a cellular derivative of MARY-X tumor explants [21]. These tumor cells form tight compact aggregates of cells termed “MARY-X spheroids” and in this study will be presented as spheroids*^MARY-X^*. These spheroids can be partitioned from the cellular debris by employing cell strainers of varying pore size. A 100 μm cell strainer (Falcon Cell Strainer; Fisher Scientific) is used to exclude spheroids of 100 μm and greater. The filtrate is then passed through a 70 μm and 40 μm cell strainer sequentially, which partitioned the 40–100 μm spheroids that were isolated and subsequently used for all drug screen analyses.

Cells were maintained in minimal essential medium (MEM) containing 10% fetal bovine serum and antibiotics (100 U/ml penicillin and 100 μg/ml streptomycin) at 37°C in an air-5% CO_2_ atmosphere at constant humidity.

All experiments were performed in compliance with the Memorial Sloan Kettering Cancer Center Animal Care and Use Program (Protocol # 06-04-006).

### Immunofluorescense

The immunofluorescent detection of P-Histone H3 and Hif1alpha were performed at the Molecular Cytology Core Facility of Memorial Sloan Kettering Cancer Center using Discovery XT processor (Ventana Medical Systems).

#### Phospho-Histone H3

The tissue sections were blocked for 30 minutes in 10% normal goat serum.2% BSA in PBS. The primary antibody incubation (rabbit polyclonal P-HH3 (Ser 10) antibody (Upstate, cat.# 06-570) was used in 1 μg/ml concentrations. The incubation with the primary antibody was done for 4 hours, followed by 32 minutes incubation with biotinylated goat anti-rabbit IgG (Vector labs, cat#:PK6101) in 7.5 μg/mL dilution. The detection was performed with Blocker D, Streptavidin-HRP D (Ventana Medical Systems), followed by incubation with Tyramide-Alexa Fluor 488 (Invitrogen, cat. #T20922).

#### Hif1 alpha

The tissue sections were blocked for 30 minutes in 10% normal goat serum. 2% BSA in PBS. The primary antibody incubation (rabbit Hif1 alpha antibody (Chemicon, cat.#AB3883) was used in 10 μg/ml concentrations. The incubation with the primary antibody was done for 5 hours, followed by 60 minutes incubation with biotinylated goat anti-rabbit IgG (Vector labs, cat#:PK6101) in 7.5 μg/mL dilution. The detection was performed with Blocker D, Streptavidin-HRP D (Ventana Medical Systems), followed by incubation with Tyramide-Alexa Fluor 594 (Invitrogen, cat. #T20935).

### Immunohistochemistry

The immunohistochemical detection of Ki67 and CAIX was performed at the Pathology Core Facility of Memorial Sloan Kettering Cancer Center.

#### Ki-67(MIB1)

Antigen recovery was conducted using heat retrieval with Citrate buffer PH6. The primary antibody incubation (Dako Cytomation, Catalog# M7240) was used at a dilution of 1:200. Standard strepavidin-biotin immunoperoxidase method and DAB as a chromogen were used.

#### Carbonic anhydrase IX (MSKCC)

Antigen recovery was conducted using heat retrieval with Citrate buffer PH6. The primary antibody incubation (MSKCC) was used at a dilution of 1:500. Standard strepavidin-biotin immunoperoxidase method and DAB as a chromogen were used.

### Confocal microscopy

Doxorubicin, a drug with intrinsic fluorescence [38], was analyzed by z-stack on a Leica TCS SP2 AOBS confocal microscope.

### Spheroids*^MARY-X^* size distribution

Spheroid size was measured using the Cellometer K2 (Nexcelom Biosciences, Lawrence, MA). A 50 μl aliquot of spheroids was loaded into the 3D chamber and assessed on the Cellometer K2. The Cellometer K2 software provides quantitative measurements of spheroid size distribution.

### Spheroids*^MARY-X^* dissolution indices

The spheroid dissolution index was determined by applying an image analysis program on ImageJ that measures the circularity of an object's area and perimeter where ‘1’ signifies a perfect circle (i.e. intact spheroid) and numbers approaching ‘0’ signify loss of circularity (i.e. dissolute spheroid). Note that ‘0’ signifies a straight line and can never be achieved in image analysis of spheroids given the innate circularity of individual cells.

### Spheroids*^MARY-X^* viability assay

Dual-fluorescent assays were performed on a Cellometer K2 (Nexcelom Biosciences, Lawrence, MA) using the viability stains, acridine orange and propidium iodide.

### Compound selection

Two representative small molecule-based libraries were used to evaluate the response of spheroids*^MARY-X^* to chemotherapeutic treatment. The first library was composed of five food and drug administration (FDA)-approved drugs that are used for treatment of both solid and blood-borne tumor types: cisplatin, doxorubicin (adriamycin), methotrexate, lapatinib and bortezomid (Figure 3). Cisplatin is a platinum-containing drug that binds to and crosslinks DNA [50]. Doxorubicin is an anthracyclin analogue that intercalates DNA [51]. Methotrexate is a folic acid antagonist that inhibits cell division by interfering with dihydrofolate reductase [52]. Lapatinib is a tyrosine kinase inhibitor that interferes with the epidermal growth factor receptor pathways [53]. Bortezomid is a modified dipeptide that acts as a proteasome inhibitor [64]. Doxorubicin, methotrexate and lapatinib are all approved for treatment of breast cancer, while doxorubicin is also recommended as adjuvant therapy in instances of nodal involvement post-surgery [55]. The drug, PU-H71, a potent inhibitor of Hsp90 was also tested. This drug was found to be efficacious in preclinical studies for triple-negative breast cancer [59] and is presently in clinical development, Phase 1: The First-in human Phase 1 Trial of PU-H71 in Patients with Advanced Malignancies (NCT01393509).

The second library was composed of two natural products of diverse chemical structure and distinctly different cellular modes of action. Taxol (paclitaxel, PTX) is a diterpene that inhibits mitosis by binding to cellular microtubules and is used for the treatment of lung, ovarian and breast cancer [65]. Gambogic acid (GA) is a natural product, derived from traditional ethnomedicine, currently in phase II clinical trials in China as an anticancer agent against non-small cell lung, colon and renal cancers [66, 67]. Compounds MAD28 and MAD44 are synthetic analogues of GA and were synthesized as previously reported [39, 60]. We have previously shown that GA, MAD28 and MAD44 localize in mitochondria to induce rapid ROS accumulation and collapse of the mitochondrial membrane potential, ultimately leading to release of cytochrome c and cell apoptosis [39]. To further drive the delivery of these compounds to mitochondria, we functionalized MAD28 and MAD44 with a triphenyl phosphonium salt [68–70]. Derivatization of these analogs with a phosphonium salt side chain was performed as described in Scheme 1. Treatment of the A-ring phenolic group with 1, 4-dibromobutane (5 equiv) under basic conditions (K_2_CO_3_) produced the corresponding alkyl bromides that, upon reaction with PPh_3_ (5 equiv) under microwave conditions produced the desired phosphonium salts CR135 and CR142 in 58% and 83% overall yields (see also supporting information for detailed experimental procedures).

### Drug screen

The 40−100 μm spheroids were distributed in equal number (~30−50 spheroids/well) to a multi-well plate. The spheroids*^MARY-X^* were treated with vehicle only (DMSO) and increasing doses of drug of interest in each 24-well plate. Following 24 hr treatment periods each well was imaged and analyzed with ImageJ for measurement of dissolution of the spheroids*^MARY-X^*. The induction of apoptosis correlates with the loss of the well circumscribed edges of the usually tight, compact spheroids*^MARY-X^* i.e. dissolution is consistent with cell death of the spheroid/embolus [27].

## SUPPLEMENTARY DATA FIGURES AND TABLE


