# Supplementary material for: Spontaneously-forming spheroids as an in vitro cancer cell model for anticancer drug screening
Source: Oncotarget. 2015 Jun 18;6(25):21255–67. doi: 10.18632/oncotarget.4013 (PMC4673263; doi:10.18632/oncotarget.4013)
Supplement: Supplementary file 1 [file oncotarget-06-21255-s001.pdf]

SUPPLEMENTARY DATA, FIGURES AND TABLE

BIOLOGICAL DATA

Spheroids^{MARY-X} size distribution, morphology and drug screening data

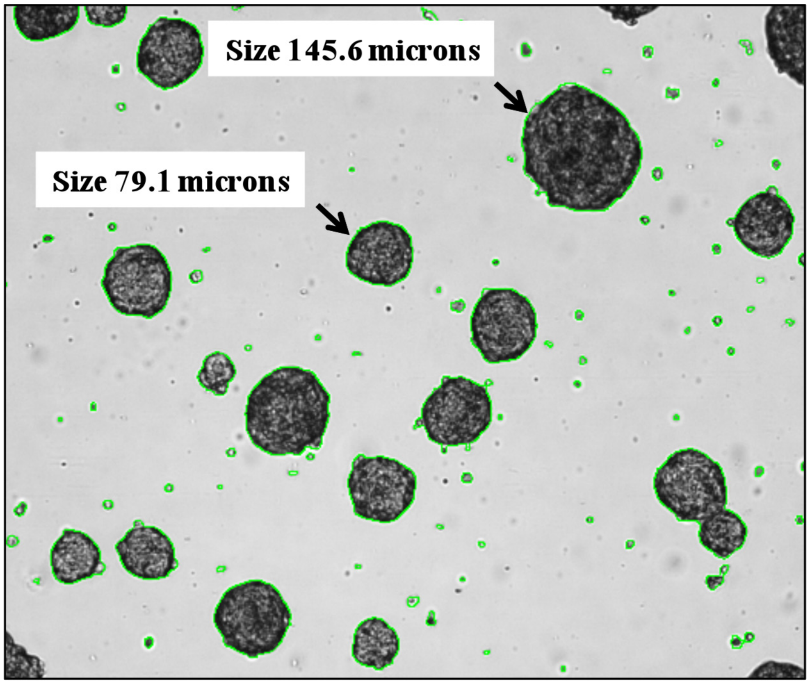

Supplementary Figure S1: Spheroids^{MARY-X} size distribution. Spheroid size was measured using the Cellometer K2 (Nexcelom Biosciences, Lawrence, MA). The Cellometer K2 software provides quantitative measurements of spheroid size distribution.

Supplementary Table S1: Response evaluation of FDA- approved drugs

FDA-Approved Drug	Response
Doxorubicin	Moderate - Complete
Bortezomib	Low - Moderate
Lapatinib	Low - Moderate
Cisplatin	No
Methotrexate	No

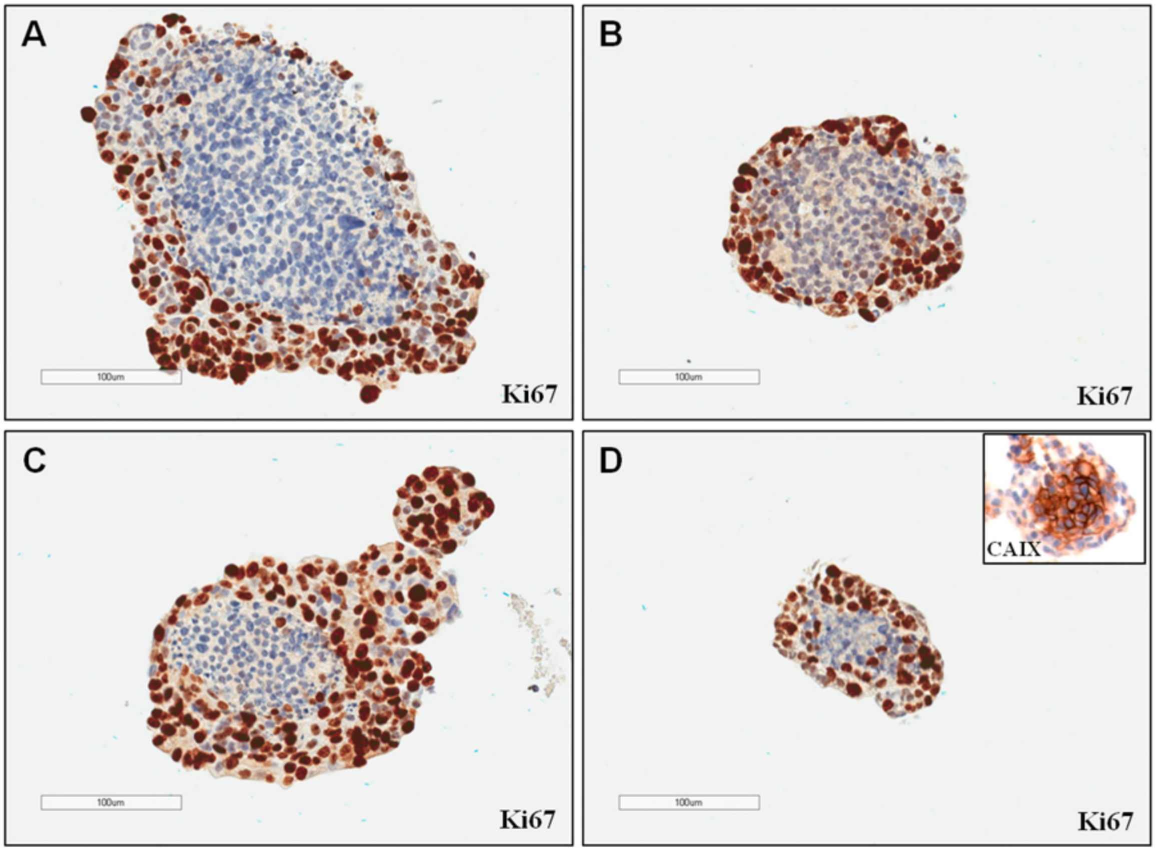

Supplementary Figure S2: Proliferative outer spheroids$^{MARY\text{-}X}$ cellular region and dormant tumor cell core. (A–D) Spheroids$^{MARY\text{-}X}$ of various sizes display consistent outer proliferative zone of highly proliferative cells when stained with Ki67 with a quiescent (i.e. dormant) core region of non-proliferative cells that (D; inset) stain positively with hypoxic marker CAIX.

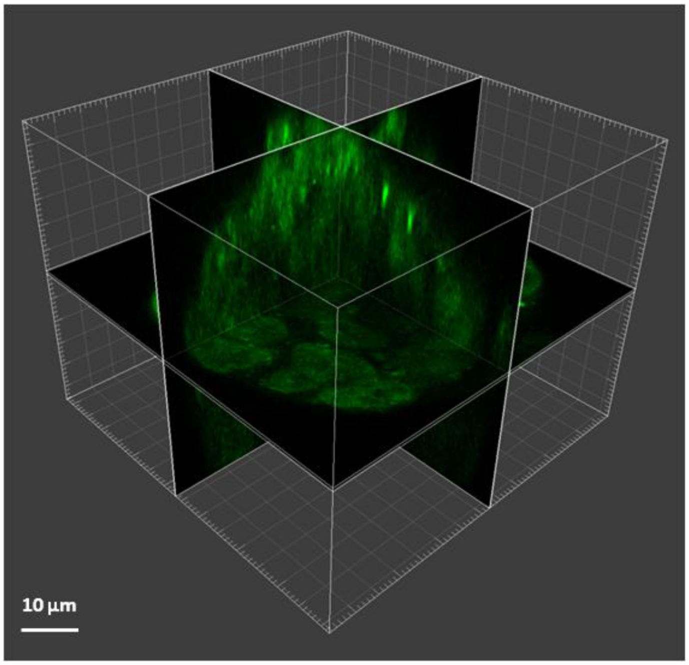

Supplementary Figure S3: Doxorubicin drug-penetration analysis. Spheroids$^{MARY\text{-}X}$ were exposed to doxorubicin for 3 hrs and then orthogonal slices presented (minus volume) following confocal microscopy z-stack analysis.

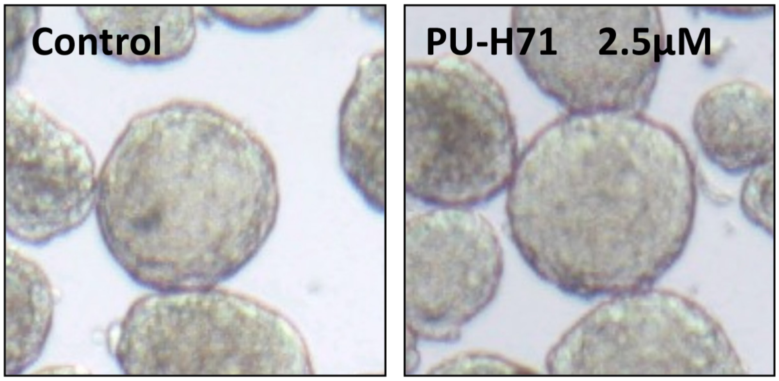

Supplementary Figure S4: PU-H71 treatment of spheroids$^{MARY\text{-}X}$. (A) Vehicle only treated spheroids$^{MARY\text{-}X}$ are indistinguishable from **(B)** PU-H71-treated with regard to well-circumscribed spheroid edges thus indicating no response to treatment.

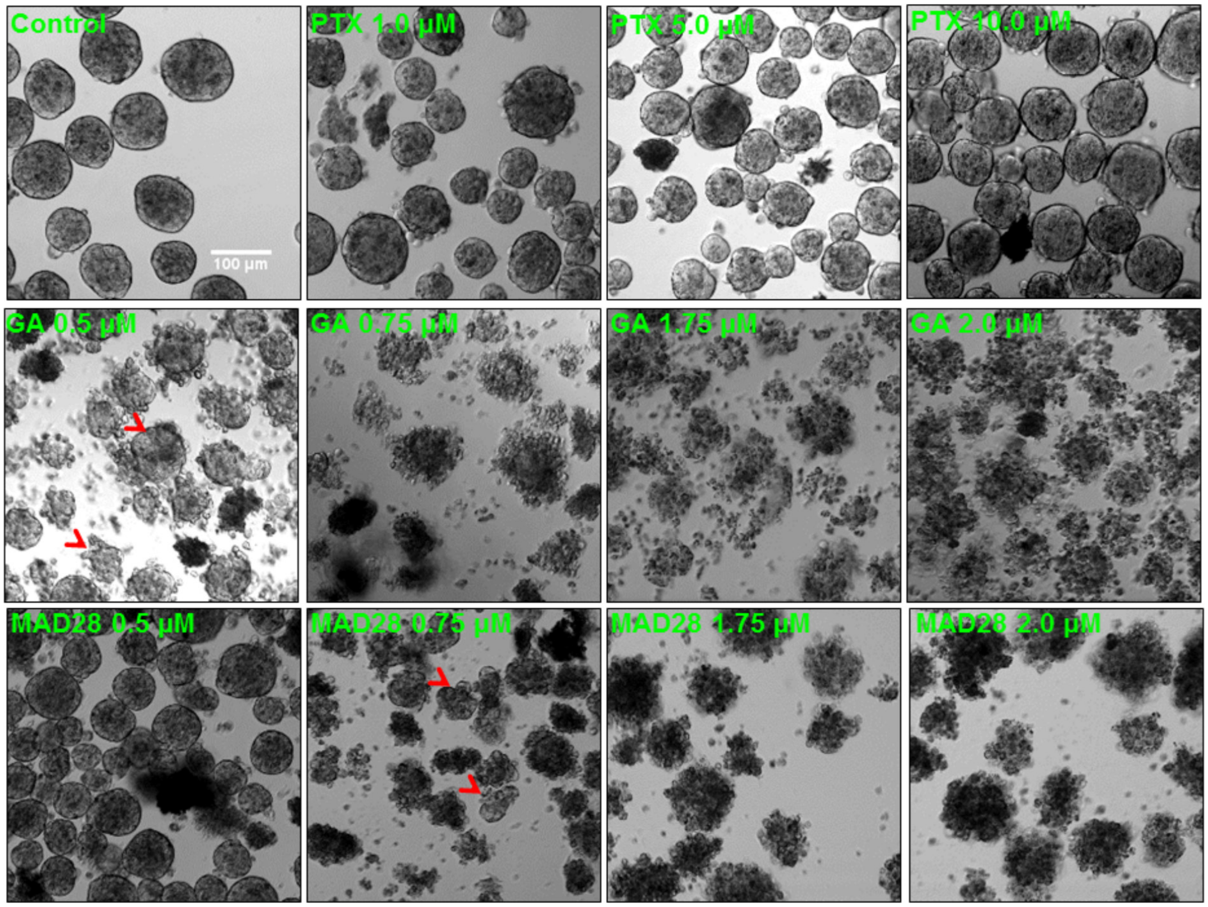

Supplementary Figure S5: Dose response analysis using spontaneously-forming spheroids$^{MARY\text{-}X}$. Gambogic acid (GA) and MAD28 display onset of response at 0.5 µM and 0.75 µM, respectively and 'complete' response as there is total dissolution of the formerly intact spheroid with well-circumscribed edges to the single cell state at 1.75 µM. (bar 100 µm)

CHEMICAL PROCEDURES

General chemical procedures

Unless indicated, all commercially available reagents were purchased at the highest commercial quality and were used as received without further purification. All nonaqueous reactions were carried out under argon atmosphere using dry glassware that had been flame-dried under a stream of argon unless otherwise noted. Anhydrous tetrahydrofuran (THF) and dimethylformamide (DMF) were obtained by passing commercially available pre-dried, oxygen-free formulations through activated alumina columns. Flash column chromatography was performed on silica gel (Merck Kieselgel 60, 230–400 mesh). The progress of all the reactions was monitored by thinlayer chromatography (TLC) using glass plates precoated with silica gel-60 F254 to a thickness of 0.5 mm (Merck), and compounds were visualized by irradiation with UV light and/or by treatment with a solution of CAM stain followed by heating. ^{1}H NMR and ^{13}C NMR spectra were recorded on a 500 MHz Varian or JEOL instrument. $CDCl_3$ was treated with anhydrous K_2CO_3, chemical shifts (δ) are quoted in parts per million (ppm) referenced to the appropriate residual solvent peak reference ($CDCl_3$), with the abbreviations s, d, t, dd, m, denoting singlet, doublet, triplet, doublet of doublets, multiplet, respectively. J = coupling constants given in Hertz (Hz). High-resolution Mass spectra (HRMS) were recorded on a trisector WG AutoSpecQ spectrometer. Gambogic acid was prepared as described in: Guizzunti, G.; Batova, A.; Chantarasriwong, O.; Dakanali, M.; Theodorakis, E. A. "Subcellular localization and activity of gambogic acid" *ChemBioChem* 2012, *13*, 1191–1198. MAD28 and MAD44 were prepared as reported in: Elbel, K. M.; Guizzunti, G.; Theodorakis, M. A.; Xu, Jing, Batova, A.; Dakanali, M.; Theodorakis, E. A. "A-ring oxygenation modulates the chemistry and bioactivity of caged *Garcinia* xanthones" *Org. Biomol. Chem.* 2013, *11*, 3341–3348.

Synthesis of CR135

Bromide 1: To a solution of MAD28 (50 mg, 0.13 mmol) in DMF (1 mL), potassium carbonate (36 mg, 0.26 mmol) and 1, 4-dibromobutane (140 mg, 0.65 mmol) were added. The mixture was left stirring at 80°C during 16 h. Upon completion, the reaction mixture was quenched with water (3 mL) and extracted with diethyl ether (2 × 10 mL). The combined organic layers were washed with brine, dried over $MgSO_4$, filtered, and concentrated in vacuum. Purification by flash column chromatography (silica, 30% EtOAc-hexane) gave bromide 1 (45.6 mg, 88.4 μmol, 68% yield). 1: Colorless liquid; *Rf* = 0.5 (50% EtOAc-hexane); ^{1}H NMR (400 MHz, $CDCl_3$) δ 7.38 (t, J = 8.4 Hz, 1H), 7.27 (m, 1H), 6.65 (d, J = 8.4 Hz, 1H), 6.54 (d, J = 8.4 Hz, 1H), 4.48 (t, J = 7.2 Hz, 1H), 4.09 (t, J = 6.0 Hz, 2H), 3.54 (t, J = 6.0 Hz, 2H), 3.46 – 3.41 (m, 1H), 2.60 (d, J = 7.7 Hz, 2H), 2.39 (d, J = 9.6 Hz, 1H), 2.32 – 2.27 (m, 1H), 2.20–2.14 (m, 2H), 2.07–2.00 (m, 2H), 1.69 (s, 3H), 1.66–1.64 (m, 1H), 1.37 (s, 3H), 1.28 (s, 3H), 1.07 (s, 3H); ^{13}C NMR (100 MHz, $CDCl_3$) δ203.61, 175.73, 161.42, 160.60, 136.63, 136.34, 134.95, 132.47, 118.81, 110.58, 110.53, 105.39, 90.06, 84.68, 83.60, 68.16, 48.70, 46.89, 34.09, 30.51, 29.93, 29.51, 29.26, 29.22, 27.76, 25.83, 25.78, 17.21; HRMS calc. for $[C_{27}H_{32}O_5Br]^+$ $(M + H)^+$ 515.1428, found 515.1426.

CR135: To a solution of 1 (35 mg, 67.9 μmol) in acetonitrile (1 mL), triphenylphosphine (89 mg, 0.34 mmol) was added. The mixture was stirred under a microwave irradiation for 2 h at 150°C. Upon completion, the reaction mixture was cooled to room temperature and the excess acetonitrile was removed by rotary evaporation. The crude was dissolved in DCM (1 mL) and hexane (10 mL) was added. The

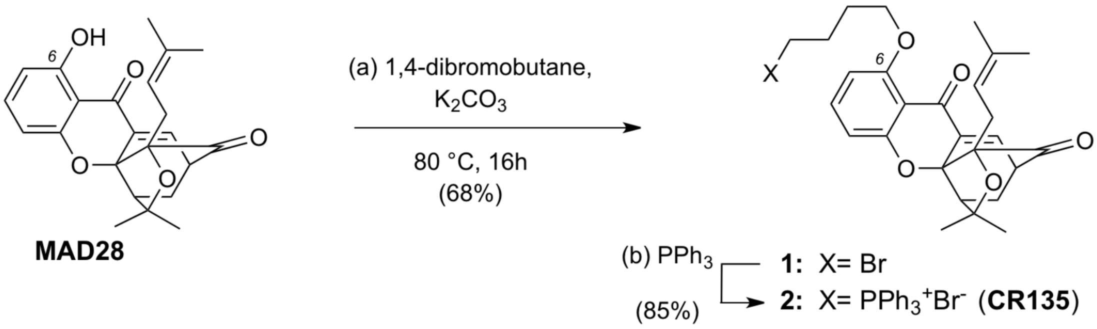

Scheme 1. *Reagents and conditions:*
(a) 1,4-Dibromobutane (5 equiv.), K_2CO_3 (2 quiv.), DMF, 80 °C, 16h, 68%;
(b) PPh_3 (5 equiv.), CH_3CN, 150 °C, microwave irradiation, 2h, 85%.

solid was filtered and washed with hexane to yield CR135 (44.9 mg, 57.7 μmol, 85% yield). CR135: White solid; *Rf* = 0.1 (20% MeOH-DCM); 1H NMR (400 MHz, CDCl3): ^{1}H NMR (400 MHz, $CDCl_3$) δ 7.88–7.66 (m, 15H), 7.42 (t, *J* = 8.4 Hz, 1H), 7.02 (d, *J* = 6.8 Hz, 1H), 6.63 (d, *J* = 8.4 Hz, 2H), 4.39 (t, *J* = 7.8 Hz, 1H), 4.21 (s, 2H), 4.11 – 4.00 (m, 2H), 3.42 (dd, *J* = 6.5, 4.4 Hz, 1H), 2.58 (d, *J* = 8.0 Hz, 2H), 2.38 (d, *J* = 9.3 Hz, 1H), 2.31 (dd, *J* = 13.2, 4.4 Hz, 1H), 2.25 (s, 2H), 1.85 (s, 2H), 1.68 (s, 4H), 1.28 (s, 3H), 1.13 (s, 3H), 1.05 (s, 3H); ^{13}C NMR (100 MHz, $CDCl_3$) δ 203.56, 175.83, 161.16, 160.36, 136.86, 135.08, 134.14, 134.04, 131.64, 130.69, 130.57, 119.25, 118.91, 118.40, 110.63, 106.01, 89.98, 84.61, 83.71, 69.05, 48.52, 46.90, 30.48, 29.29, 29.19, 26.02, 25.74, 22.69, 22.19, 20.43, 17.24; HRMS calcd for $[C_{45}H_{46}O_5P]^+$ (M-Br)$^+$ 697.3077, found 697.3074.

Synthesis of CR142

Bromide 3: To a solution of MAD44 (0.1g, 0.26 mmol) in DMF (2 mL), potassium carbonate (72 mg, 0.52 mmol) and 1, 4-dibromobutane (0.28 g, 1.31 mmol) were added. The mixture was left stirring at room temperature during 8 h. Upon completion, the reaction mixture was quenched with water (10 mL) and extracted with diethyl ether (2 × 20 mL). The combined organic layers were washed with brine, dried over $MgSO_4$, filtered, and concentrated in vacuo. Purification by flash column chromatography (silica, 30% EtOAc-hexane) gave 3 (0.11 g, 0.22 mmol, 85% yield). 3: Colorless liquid; $_{Rf}$ = 0.5 (30% EtOAc-hexane); ^{1}H NMR (400 MHz, $CDCl_3$) δ 7.88 (d, *J* = 8.8 Hz, 1H), 7.38 (d, *J* = 7.0 Hz, 1H), 6.61 (dd, *J* = 8.8, 2.1 Hz, 1H), 6.45 (d, *J* = 2.1 Hz, 1H), 4.44 (t, *J* = 7.4 Hz, 1H), 4.06 (t, *J* = 5.9 Hz, 2H), 3.52 – 3.45 (m, 3H), 2.60 (d, *J* = 8.3 Hz, 2H), 2.43 (d, *J* = 9.5 Hz, 1H), 2.32 (dd, *J* = 13.5, 4.6 Hz, 1H), 2.10 – 2.05 (m, 2H), 2.02 – 1.96 (m, 2H), 1.71 (s, 3H), 1.66 (s, 1H), 1.34 (s, 3H), 1.25 (s, 3H), 1.00 (s, 3H); ^{13}C NMR (100 MHz, $CDCl_3$) δ 203.53, 175.45, 165.83, 161.84, 135.04, 134.94, 133.15, 129.06, 118.81, 113.33, 111.05, 101.49, 90.98, 84.76, 83.65, 77.58, 77.26, 76.94, 67.68, 49.03, 46.91, 33.42, 32.16, 30.65, 29.94, 29.47, 29.30, 27.84, 25.64, 25.47, 22.93, 17.15, 14.37; HRMS (ESI) m/e 515.1428 [M+H]$^+$ calcd for $[C_{27}H_{32}BrO_5]^+$: 515.1428.

CR142: To a solution of **3** (0.1 g, 0.19 mmol) in acetonitrile (5 mL), triphenylphosphine (0.25 g, 0.97 mmol) was added. The mixture was stirred under a microwave irradiation for 2 h at 150°C. Upon completion, the reaction mixture was cooled to room temperature and the excess acetonitrile was removed by rotary evaporation. The crude was dissolved in DCM (3 mL) and hexane (30 mL) was added. The solid was filtered and washed with hexane to yield CR142 (0.15 g, 0.18 mmol, 98% yield). CR142: White solid; R_f = 0.1 (20% MeOH-DCM); ^{1}H NMR (400 MHz, $CDCl_3$)δ 7.89–7.77 (m, 10H), 7.71–7.67 (m, 5H), 7.37 (d, *J* = 7.0 Hz, 1H), 7.17 (d, *J* = 8.8 Hz, 1H), 6.51 (dd, *J* = 8.8, 2.2 Hz, 1H), 6.40 (d, *J* = 2.2 Hz, 1H), 4.43 (t, *J* = 7.2 Hz, 1H), 4.20 – 4.11 (m, 2H), 4.03 (dd, *J* = 16.8, 12.9 Hz, 2H), 3.46 (dd, *J* = 6.5, 4.5 Hz, 1H), 2.58 (d, *J* = 7.8 Hz, 2H), 2.42 (d, *J* = 9.5 Hz, 1H), 2.35 (s, 1H), 2.33–2.24 (m, 2H), 1.87 (s, 2H), 1.71 (s, 4H), 1.31 (s, 3H), 1.28 (s, 3H), 0.96 (s, 3H); ^{13}C NMR (100 MHz, $CDCl_3$) δ 203.54, 175.46, 165.76, 161.82, 135.27, 135.06, 134.84, 134.01, 133.91, 133.12, 131.13, 130.78, 130.66, 128.97, 118.95, 118.09, 113.31, 111.02, 101.70, 90.96, 84.73, 83.71, 68.36, 67.53, 48.98, 46.92, 38.92, 30.72, 30.56, 29.24, 29.13, 25.64, 25.44, 23.94, 23.21, 19.49, 17.17, 14.30, 11.19; HRMS (ESI) m/e 697.3077 [M-Br]$^+$ calcd for $[C_{45}H_{46}O_5P]^+$: 697.3075.

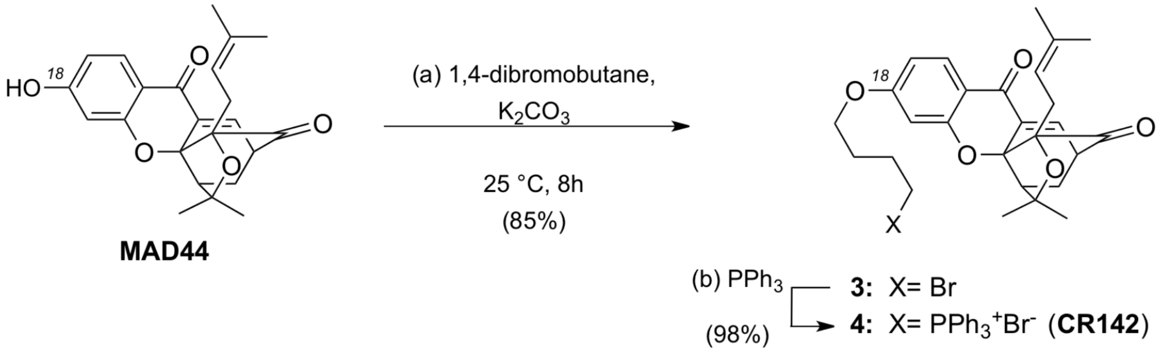

Scheme 2. *Reagents and conditions:*
(a) 1,4-Dibromobutane (5 equiv.), K_2CO_3 (2 quiv.), DMF, 25 °C, 8h, 85%;
(b) PPh_3 (5 equiv.), CH_3CN, 150 °C, microwave irradiation, 2h, 98%.

NMR Spectra

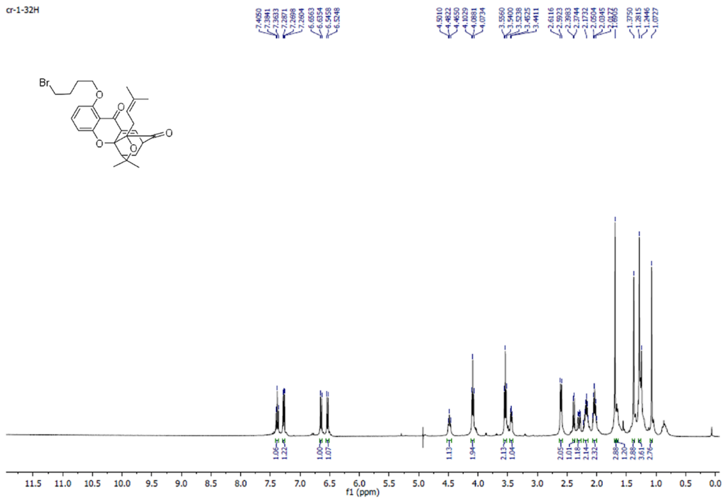

^{1}H NMR (500 MHz, $CDCl_3$) of **1**.

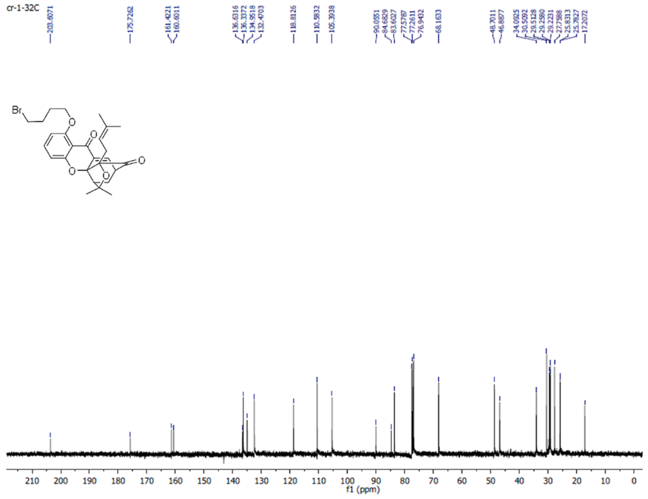

^{13}C NMR (125 MHz, $CDCl_3$) of **1**.

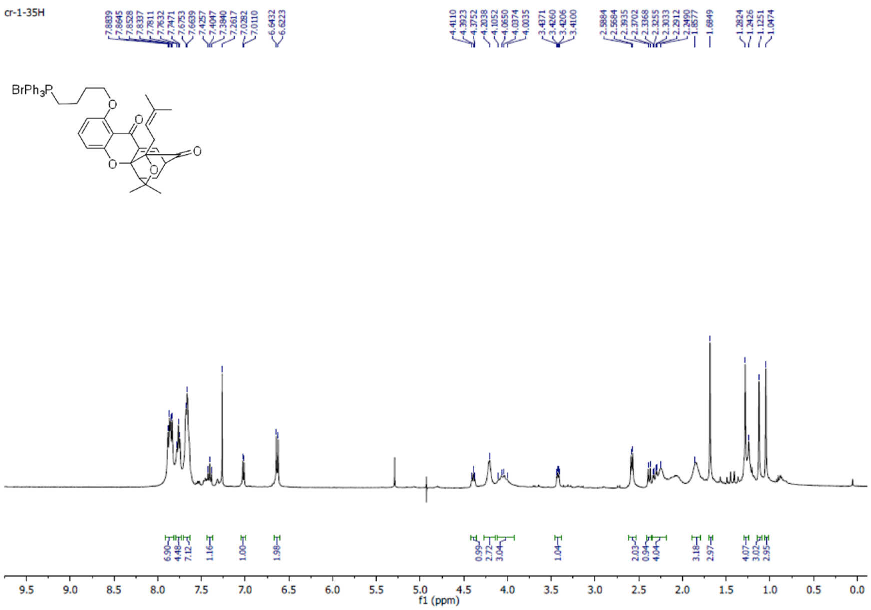

^{1}H NMR (500 MHz, $CDCl_3$) of **CR135**

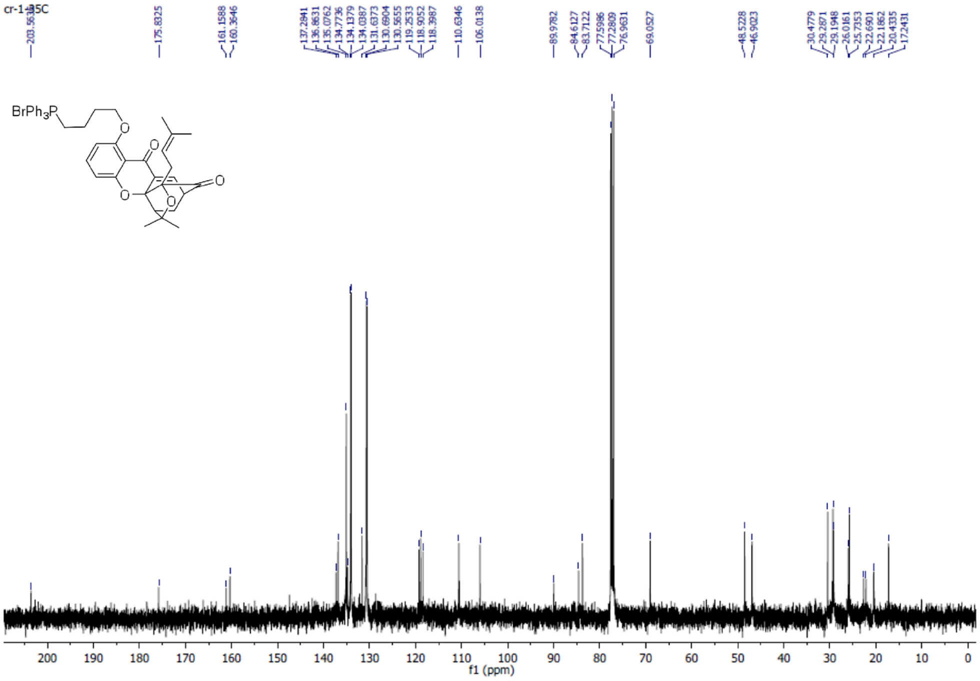

^{13}C NMR (125 MHz, $CDCl_3$) of **CR135**.

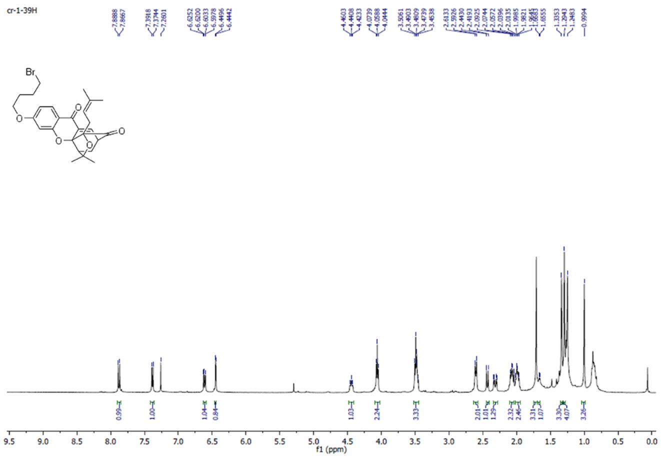

^{1}H NMR (500 MHz, $CDCl_3$) of **3**.

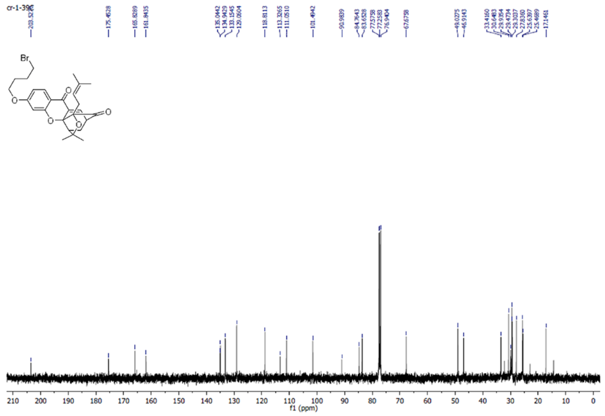

^{13}C NMR (125 MHz, $CDCl_3$) of **3**.

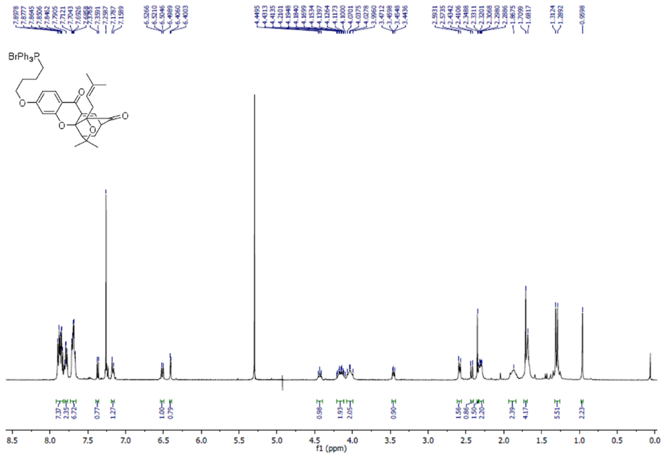

^{1}H NMR (500 MHz, $CDCl_3$) of **CR142**.

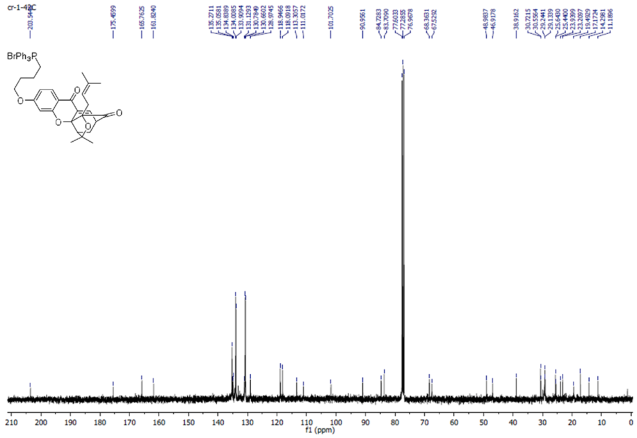

^{13}C NMR (125 MHz, $CDCl_3$) of **CR142**.
